# Efficacy of Smear Layer Removal of Human Teeth Root Canals Using Herbal and Chemical Irrigants: An In Vitro Study

**DOI:** 10.7759/cureus.40467

**Published:** 2023-06-15

**Authors:** Mridusmita Mukherjee, Tribisha Kalita, Pranamee Barua, Atrayee Barman, Salouno Thonai, Putul Mahanta, Himchumi Medhi

**Affiliations:** 1 Conservative Dentistry & Endodontics, Government Dental College, Silchar, IND; 2 Conservative Dentistry & Endodontics, Siddhartha Dental Clinic and Implant Centre, Guwahati, IND; 3 Pediatric & Preventive Dentistry, Regional Dental College, Guwahati, IND; 4 Orthodontics & Dentofacial Orthopedics, Government Dental College, Dibrugarh, IND; 5 Conservative Dentistry & Endodontics, Regional Dental College, Guwahati, IND; 6 Forensic Medicine and Toxicology, Nalbari Medical College and Hospital, Nalbari, IND

**Keywords:** scanning electron microscope, root canal therapy, herbal irrigants, dentin, sodium hypochlorite

## Abstract

Objectives

Over many years, many intracanal irrigants have removed smear layers during routine root canal therapy. The efficacies of conventional endodontic chemical irrigants are documented, but limited research is available on herbal irrigants' role in the endodontic therapy irrigation protocol. This study aimed to evaluate endodontic irrigants' smear layer removal efficacy, namely, 3% sodium hypochlorite (NaOCl), 17% ethylenediaminetetraacetic acid (EDTA), green tea extract, and Triphala extract, using scanning electron microscopy (SEM) analysis.

Methods

Fifty freshly extracted human permanent maxillary and mandibular single-rooted teeth were collected from the Oral Surgery Department of Regional Dental College (RDC), Guwahati, India. The samples were divided into five groups with 10 teeth each: Group A: sterile distilled water (negative control), Group B: 3% NaOCl, Group C: 17% EDTA, Group D: green tea, and Group E: Triphala (citric acid). Each tooth was then longitudinally split and prepared for SEM inspection under 1000X magnification. The comparison of smear layer removal scores between the groups was done by the Kruskal-Wallis test and Mann-Whitney test, with the significance level set at p<0.01.

Results

The comparison of the overall mean smear scores and those at different root portions shows that Group C has the lowest mean score, followed by Group E. The pairwise comparison shows that the difference in the mean smear scores between Group C and the other four groups is statistically significant (p-value<0.05). Moreover, the difference in the overall, coronal, middle, and apical mean smear scores between Group A and Group E was highly significant (p-value<0.001).

Conclusions

The highest smear layer removal efficacy was observed in the samples treated with 17% EDTA. Moreover, the clearing efficacy of Triphala is significantly better than that of distilled water in smear layer removal.

## Introduction

Endodontics is primarily a clinical field that prevents and controls root canal infections, and the complexity of the root canal morphology presents a challenging objective for the endodontics community. Although the rotary instrument technology has advanced recently, mechanical instrumentation alone cannot completely eliminate microorganisms from the root canal structure [1]. To achieve the kind of debridement that can promote the adhesion of restorative materials to the root canal, suitable irrigants are used during biomechanical preparation [2].

For effective root canal cleaning, irrigation and instrumentation are used. Massive amounts of dentin waste combined with remaining healthy and necrotic pulp tissues during instrumentation create a smear layer that is glued to the root canal wall by microorganisms and microbiological contaminants [3]. If retained, this layer can affect the dissemination of irrigants and hence can compromise the treatment outcome. As the smear layer contains both organic and inorganic components, such as blood, bacteria, and leftovers of odontoblastic processes, removing it is necessary and highly preferred [4].

Various irrigants, such as sodium hypochlorite, hydrogen peroxide, ethylenediaminetetraacetic acid (EDTA), and chlorhexidine, are used to remove the smear layer. The quality of obturation may be improved by the irrigants' ability to completely remove all canal debris [5]. An irrigant's job involves both the debridement of the smear layer and the dissolution of necrotic and vital tissues.

Although the efficacy of conventional endodontic chemical irrigants has been proven, they have various disadvantages, such as instrument corrosion, unpleasant taste, damage to the surrounding tissues, and incapability to completely remove the smear layer. This has directed the testing of various herbal endodontic irrigants. Herbal irrigants are cost-effective, less toxic, and better tolerated by patients. Natural substances, including herbal extracts, are becoming increasingly significant as endodontic irrigants as those contain active components with antioxidant, antimicrobial, anti-inflammatory, immune-enhancing, and other favorable properties [6]. Provided that the efficacies of both types of irrigants are comparable, herbal irrigants may be well thought-out as a substitute for chemical irritants soon. Therefore, this study compared the efficacy of smear layer removal of root canals using various herbal and chemical irrigants. The present research uses scanning electron microscopy (SEM) analysis to evaluate endodontic irrigants' smear layer removal efficacy, namely, 3% sodium hypochlorite (NaOCl), 17% EDTA, green tea extract, and Triphala extract.

## Materials and methods

The present cross-sectional comparative study included 50 freshly removed human permanent maxillary and mandibular single-rooted teeth collected from the Oral Surgery Department of Regional Dental College, Guwahati, India. The collected teeth were properly washed with running tap water to remove any blood and debris from the surface. The samples were stored in a saline solution.

The samples were divided randomly into five groups with 10 teeth each. They were organized according to the irrigants used: Group A: sterile distilled water (negative control), Group B: 3% NaOCl, Group C: 17% EDTA, Group D: green tea, and Group E: Triphala (citric acid). NaOCl is widely recommended as an irrigant because of its microbial and organic tissue-dissolving ability. The irrigants used in Group D comprised green tea polyphenols obtained from shoots of *Camellia sinensis*, containing carotenoids, vitamin C, and minerals. Triphala, a dried powder composed of three medicinal herbs, was used in group E. It is comprised of the fruits of the* Emblica officinalis* (Amalaki), *Terminalia bellerica* (Bibhitaki), and *Terminalia chebula *plants (*Haritaki*) [7].

Final rinses were used during and after the instrumentation. The working length was measured radiographically to be 1 mm short of the apical foramen. A size 15 K-file was used to generate the glide path (Mani Inc., Japan). According to the manufacturer's specifications, the root canals were prepared in a crown-down manner using ProTaper Gold rotary system (Dentsply Sirona, United States) at 300 rpm speed and 5.10 N cm torque. The canals were irrigated using 5 mL of the prepared solutions corresponding to the respective group during the instrumentation. The study samples were subsequently rinsed with sterile distilled water in each group and dried with sterile absorbent paper sheets (Diadent Group International, Canada). The samples were then stored in 100% humidity for one week at 37 °C in an incubator.

Afterward, the roots were grooved vertically on the buccal and lingual surfaces using a diamond bur, while avoiding to touch the canal wall. After that, each root was divided in the buccolingual direction with a chisel and mallet. By using a sharp knife to make tiny grooves on the side of the root, each sample was split into three equal portions the apical, middle, and coronal thirds. The sections were evaluated, and the cleaner halves were selected to standardize the result. The selected dentinal surfaces were marked at 3 mm, 6 mm, and 9 mm from the apical end and were viewed at a point equidistant from the lateral walls. The samples were dehydrated by a series of graded ethanol solutions and then coated with a gold layer, after which they were evaluated using a scanning electron microscope (JEOL JSM-6360, Japan) at 1000X magnification. Attur et al. described a technique to standardize the area examined for each sample [8].

The collected data were subjected to the Kruskal-Wallis H test to compare the mean smear scores of the five groups at different root levels and the Mann-Whitney test to compare the mean smear scores between the groups, considering p<0.05 as statistically significant.

The ethics committee of Regional Dental College, Guwahati, India, has given the ethical clearance with approval no. RDC/29/2011/1243.

## Results

The comparison of the overall mean smear scores of the groups, irrespective of the root portion, shows that the group with the least mean score was Group C (1.09±0.43), followed by Group E, Group B, and Group D. The maximum mean smear score was noted in the control group (Group A). The Kruskal-Wallis H test revealed a significant variation in the overall mean smear scores among the five groups. Moreover, when the mean smear scores of the five groups were compared at the coronal, middle, and apical levels of the root, Group C had the least mean scores at all three root levels, followed by Group E, Group B, and Group D. The highest mean score was observed in Group A. The mean smear scores were found to differ significantly (p-value<0.001) among the groups at various root levels also (Table 1).

**Table 1:** Kruskal-Wallis H test for the comparison of the mean smear scores of the five groups at different root levels #p-value for Kruskal-Wallis H test; Group A: sterile distilled water; Group B: 3% NaOCl; Group C: 17% EDTA; Group D: green tea; Group E: Triphala; Std Dev: standard deviation

Group	Overall	Coronal	Middle	Apical
	Mean ± Std Dev	Mean ± Std Dev	Mean ± Std Dev	Mean ± Std Dev
A	2.68±0.48	2.84±0.66	2.53±0.68	2.68±0.61
B	1.57±0.46	1.76±0.48	1.85±0.51	1.64±0.40
C	1.09±0.43	1.15±0.43	1.03±0.44	1.10±0.41
D	2.12±0.71	2.00±0.72	2.2±0.76	2.16±0.65
E	1.55±0.43	1.62±0.41	1.57±0.43	1.45±0.45
p-value^#^	<0.0001	<0.0001	<0.0001	<0.0001

The pairwise comparison of the overall mean smear scores revealed that Group C's mean smear score significantly differed from those of the other four groups at various root levels (p-value<0.05). The mean smear score of Group C also significantly differed from those of the other four groups at various root levels (p-value<0.05). Thus, the clearing efficacy of EDTA in the smear layer removal was substantially different from all the other irrigants at the overall, coronal, middle, and apical root levels. The findings indicate that the clearing efficacy of EDTA in smear layer removal is significantly better than those of distilled water (p-value<0.001), sodium hypochlorite (p-value<0.05), Triphala (p-value<0.05), and green tea. Moreover, the group-wise evaluation revealed that extremely significant differences (p-value<0.001) exist in the mean smear scores of Group A and Group E at different root levels. Thus, the effectiveness of Triphala in smear layer removal was notably better than that of distilled water. The findings suggest that Triphala and EDTA are equally efficient in eradicating the smear layer from root canals. No significant variability was noted in the cleaning efficiencies of distilled water, green tea, and sodium hypochlorite (Table 2).

**Table 2:** Mann-Whitney U test for the inter-group comparison of the mean smear scores ##p-value for Mann-Whitney U test; Group A: sterile distilled water; Group B: 3% NaOCl; Group C: 17% EDTA; Group D: green tea; Group E: Triphala

Between-group comparisons	Overall	Coronal	Middle	Apical
	p-value^##^	p-value^##^	p-value^##^	p-value^##^
A vs. C	p<0.001	p<0.001	p<0.001	p<0.001
A vs. E	p<0.001	p<0.001	p<0.001	p<0.001
B vs. C	0.026	0.020	0.018	0.02
D vs. C	0.001	0.004	p<0.001	p<0.001
E vs. C	0.027	0.022	0.012	0.034

**Figure 1:**
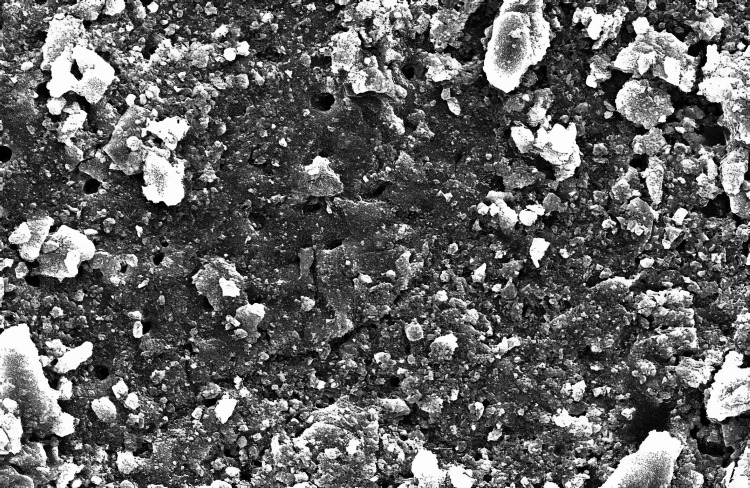
Scanning electron microscopic image of Group A

The SEM-evaluated images using 1000X magnification of the five groups studied are as follows (Figure 1):

**Figure 2:**
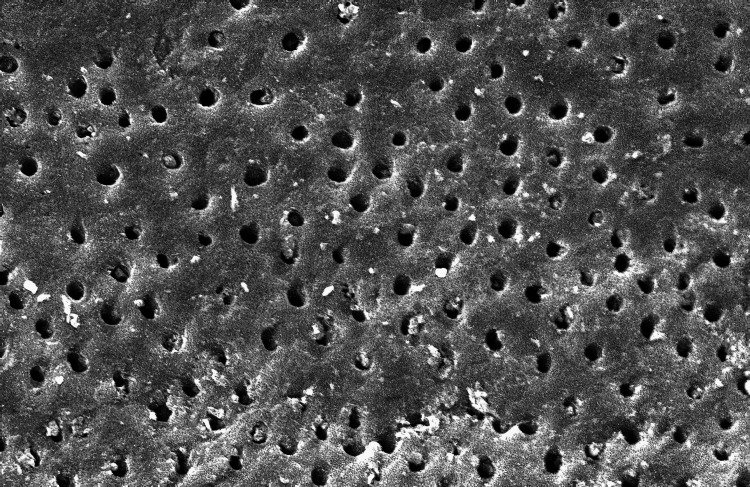
Scanning electron microscopic image of Group B

**Figure 3:**
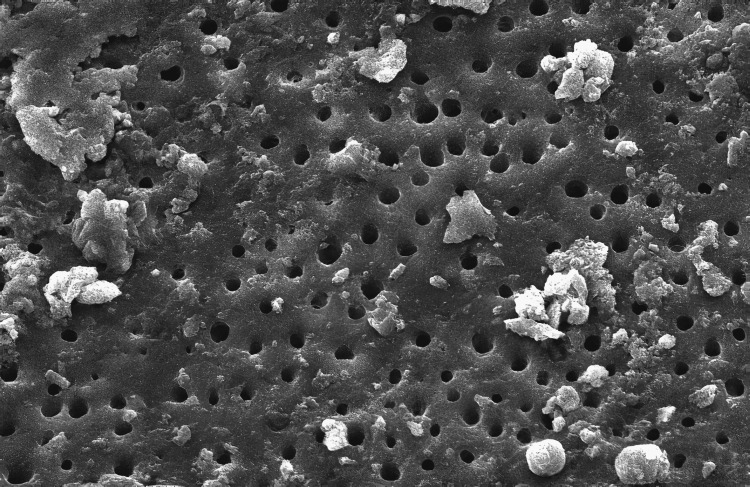
Scanning electron microscopic image of Group C

**Figure 4:**
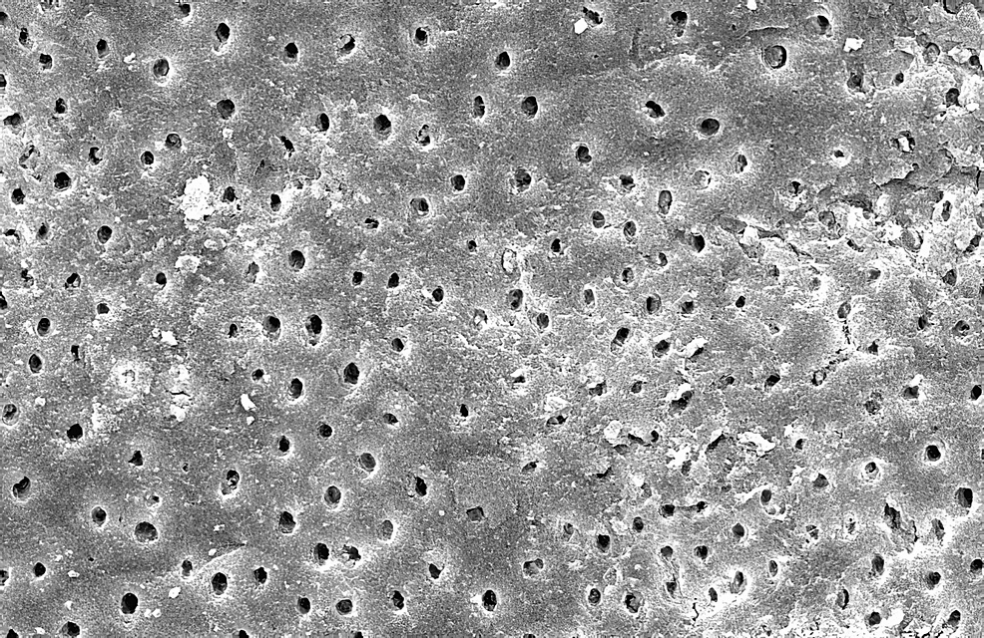
Scanning electron microscopic image of Group D

**Figure 5:**
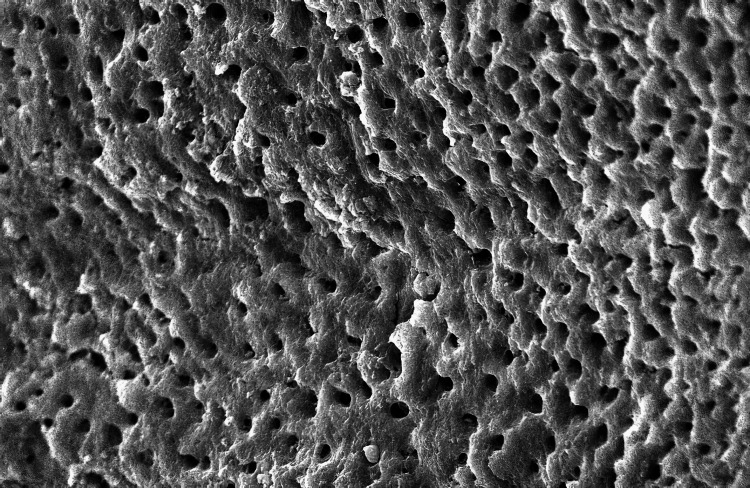
Scanning electron microscopic image of Group E

## Discussion

Smear layer removal is essential in endodontic therapy as it contains inflamed necrotic tissue, microbes or biofilms, and other debris. The persistence of the smear layer may alter the microbial community and invite reinfection [5]. If not eliminated, it can hinder the passage of irrigation regimens.

In Group A (control group), smear plugs covered the dentinal tubules. Among the experimental groups, in Group B, where 3% NaOCl was used, the dentinal tubules were entirely covered by the smear layer in all halves of the root canal space. These findings aligned with those of a related investigation [9]. Although NaOCl is widely recommended because of its microbial and organic tissue-dissolving ability, it still does not effectively remove the smear layer. When a sodium hypochlorite solution is extruded via the apex at clinically advised dosages, numerous ex vivo and in vivo experiments have documented mild to severe cytotoxicity [10,11]. In Group C, there was an incomplete removal of the smear layer in the third apical area, which agrees with similar other findings [12-14].

In Groups D and E, dimethyl sulfoxide was used as an explicit polar solvent for Triphala and green tea polyphenols [15,16]. This was stirred for two minutes and passed through a fast filter paper. In the Group D samples, no smear plugs were present in the coronal and middle thirds, which is evident in the apical segment. On the contrary, the samples in Group E showed clear dentinal tubules in the coronal and middle thirds, lesser in the apical thirds. The reason may be the presence of citric acid in the herbal preparation, which is abundant in* Emblica officinalis* and is responsible for its astringent property [17,18]. Similar results were also demonstrated by other studies, which concluded that Triphala has a good cleaning efficacy compared to distilled water, green tea, and 2% hypochlorite. However, the most effective smear layer removal occurred with sodium hypochlorite with a final rinse of 17% EDTA, followed by Triphala [19]. 

Furthermore, another study that compared the smear layer removal ability of different solutions of Triphala when used in a specific irrigant protocol concluded that the use of Triphala as a final rinse solution during endodontic therapy seems promising [20].

The present study observed a considerable disparity in the overall mean smear scores among the five groups. The mean smear scores were lowest in Group C at different root regions, followed by Groups E, B, D, and A. The mean smear scores of the five groups differed significantly (p-value<0.001) at various root levels. The overall mean smear score of Group C was notably different from those of the other four groups (p-value<0.05). The mean smear score of Group C also significantly differed from those of the other four groups (p-value<0.05) at different root regions. The observations indicate the better clearing efficacy of EDTA in smear layer removal than the other irrigants. Recent research has also suggested that EDTA outperforms herbal medicines in eliminating smear layers, which is in concordance with our findings [21,22]. Although it is vital in eliminating the smear layer, EDTA tends to cause dentinal erosion and increases the risk of perforation during instrumentation, depending on the concentration volume and contact time [23].

In addition, the group-wise analyses revealed substantial variations (p-value<0.001) between Group A and Group E's mean smear scores at distinct root areas. The findings suggest that Triphala is as effective as EDTA in smear layer removal from root canals. Triphala is a highly effective polyherbal Ayurvedic medication. Gallic acid, vitamin C, quinones, flavones, flavonoids, and flavonols are just a few of the components found in Triphala formulations, all of which contribute to the pharmacological and physiological effects of the plant [7]. As an oral rinse solution, Triphala extract reduces gingival inflammation without side effects [24]. It has been established that Triphala is efficient against *Enterococcus faecalis* biofilms and possesses antifungal qualities [25-26]. Comparable to our findings, various studies agreed that Triphala is as productive as other chemical irrigants in smear layer removal [25-27].

Limitation

The present study is in vitro in nature. Further long-term in vivo biocompatible studies and clinical trials are needed to evaluate herbal irrigants' sufficient properties so that those can be confidently used in endodontics as irrigating solutions.

## Conclusions

Triphala is as effective as EDTA in removing the smear layer from root canals during endodontic procedures. Herbal irrigants like Triphala may be encouraged and advocated in smear layer removal procedures due to their comparable efficacy to chemical irrigants and lesser side effects. Awareness should be created among clinicians regarding the effectiveness of herbal irrigates in adequate smear layer removal, which can ensure a better outcome in root canal therapy.
